# Virtual Reality–Enabled Physical AI Training for Supportive Nursing Robotics: Nurse-in-the-Loop, Site-Specific Conceptual Framework

**DOI:** 10.2196/96848

**Published:** 2026-10-05

**Authors:** Seungman Kim

**Affiliations:** 1 School of Nursing, Texas Tech University Health Sciences Center, 3601 4th Street, Lubbock, TX, 79430, United States, 1 8067815683

**Keywords:** virtual reality, physical AI, nursing robotics, digital twin, simulation-based learning, nurse augmentation, human-in-the-loop learning, simulation-to-real transfer, nursing informatics

## Abstract

Interest in physical AI and robotics in health care is increasing, but the nursing literature shows that the evidence base remains early, nurse-centered applications are underdeveloped, and real-world experiential evidence is limited. Nurses are more likely to accept robots that reduce physically demanding and repetitive work while preserving the interpersonal and judgment-intensive core of nursing practice. This conceptual paper proposes a nursing-centered framework in which virtual reality functions not merely as a simulator but as a scaffolded training infrastructure for supportive physical AI systems, enabling nurse augmentation, site-specific adaptation through digital twins, and staged simulation-to-real transfer. The framework was developed through a conceptually integrative and implementation-aware synthesis drawing on nursing robotics, AI in nursing, immersive simulation, digital twins, human-in-the-loop learning, and physical AI development literature. It is organized around 5 linked elements and supported by a 4-layer technical architecture that outlines functional requirements and implementation pathways. Four key propositions ground the framework: (1) nursing robot training should focus on competency formation, not on decontextualized data accumulation; (2) training should proceed through progressive fidelity and staged autonomy; (3) digital twins should function as operational bridges for local ward adaptation; and (4) simulation-to-real transfer should be governed by explicit nursing-relevant validation criteria and retained human accountability. The proposed nurse-in-the-loop, site-specific virtual reality framework offers a nursing-centered complement to general-purpose physical AI pipelines by making workflow fit, role boundaries, local adaptation, and governed transfer explicit design requirements.

## Introduction

AI in nursing has advanced rapidly in decision support, workflow optimization, and documentation, yet embodied AI for nursing remains underdeveloped as a clinically grounded field. Recent umbrella and scoping reviews show unresolved ethical, social, and implementation questions, while robotics reviews suggest that the evidence base is early and that explicitly nurse-centered robotic support remains limited [1,2]. Shaw and Chen [3] identify physical AI as a nascent but consequential frontier for nursing, emphasizing that robotics applications must be evaluated through nursing-specific lenses rather than imported wholesale from industrial or general-purpose contexts. Qualitative synthesis further confirms that nurses perceive AI adoption as acceptable only when it preserves professional autonomy and relational care [4].

Nursing work is not simply a set of motor actions. It is dynamic, interruption-prone, socially dense, ethically constrained, and deeply local. A single nurse on a medical-surgical unit may manage 6 or more patients simultaneously, responding to unpredictable deteriorations, coordinating with multiple disciplines, and making real-time prioritization decisions that cannot be reduced to algorithmic rules. Whether a robot can function appropriately in this context depends not only on navigation or manipulation capabilities but also on understanding role boundaries, fitting into existing workflows without creating new burdens, supporting appropriate escalation when uncertainty arises, and preserving the human clinical judgment that remains the irreducible core of safe patient care [5,6]. These requirements are not peripheral constraints to be addressed after technical development; they are the design specifications that should drive development from the outset.

Industry-scale physical AI platforms such as Cosmos (NVIDIA) and Isaac Sim (NVIDIA) provide powerful enabling technologies—world foundation models, synthetic data generation, physically based simulation—that future nursing robotics will leverage. These platforms have demonstrated remarkable progress in warehouse logistics, manufacturing, and autonomous driving, where environments are relatively structured and task specifications can be defined independently of individual human relationships. However, these platforms are optimized for generalizable autonomy at scale, whereas nursing requires an additional nurse-centered orchestration layer that addresses workflow fit, role clarity, safety governance, and local adaptation as first-class design requirements. The gap is not primarily technological; it is translational. The enabling components exist, but the orchestration layer that connects them to nursing’s specific clinical, ethical, and organizational realities does not.

This paper proposes a nurse-in-the-loop virtual reality (VR) framework with site-specific digital twin alignment and governed simulation-to-real transfer. The framework contributes both a conceptual structure and a practical technical architecture, the latter detailed in Multimedia Appendix 1 to support the assessment of conceptual plausibility and future empirical implementation.

The framework was developed through a conceptually integrative synthesis rather than a systematic review, and the procedure is made explicit here for transparency. Source literature was identified purposively across 6 domains—nursing robotics, AI in nursing, immersive simulation, digital twins, human-in-the-loop learning, and physical AI development—prioritizing peer-reviewed work, recent reviews and authoritative reports (including systematic reviews, umbrella reviews, and a National Academies report), and sources judged relevant to the translational question of how nursing expertise can govern robot learning. This selection was purposive and interpretive rather than exhaustive or reproducible in the sense of a systematic search protocol. Synthesis proceeded thematically. Recurring requirements and constraints were extracted across these domains and grouped into the dimensions that structure the framework: task decomposition, expert-in-the-loop learning, fidelity progression, local adaptation, and governed transfer. Conceptual abstraction then moved from specific technologies and empirical findings to architecture-level principles, distilling the recurring patterns into the 4 propositions that ground the framework and into the 5 linked elements and 4-layer architecture that operationalize them. The framework is therefore a structured, literature-grounded hypothesis whose components are intended to be tested empirically rather than a set of validated findings.

## Why Nursing Requires a Distinct Physical AI Training Paradigm

Reviews consistently show nurses prefer robots that reduce physically burdensome repetitive work while preserving interpersonal and judgment-intensive care [7]. A systematic review of collaborative robots found that most applications are patient-centered rather than nurse-centered, and an umbrella review identified persistent concerns around patient safety, liability, and ethics [1,2]. This pattern reveals a fundamental misalignment in how nursing robotics has been conceived: robots are designed for tasks defined by engineers or physicians, then offered to nurses as end users, rather than developed from the outset around nurse-identified needs and nurse-defined boundaries. The consequence is a literature rich in technical demonstrations but thin on evidence of sustained clinical adoption, workflow integration, or measurable workload relief for nursing staff.

The relevant design question is therefore not “How can we maximize robotic autonomy?” but “Which parts of nursing work are appropriate for bounded robotic support, under which workflow and safety constraints, and with what forms of nurse oversight?” This reframing has several implications. It shifts the unit of analysis from the robot’s capabilities to the nurse’s workflow. It requires that task selection be informed by practicing nurses rather than inferred from external observation. It demands that autonomy levels be calibrated to clinical risk rather than maximized for technical elegance, and it positions evaluation around nursing-relevant outcomes, including workload relief, trust, care quality, and escalation appropriateness, rather than task success rates in isolation. Collectively, these shifts define a translational agenda that positions nurses as architects of robotic scope rather than recipients of autonomous systems.

## A Nurse-in-the-Loop, Site-Specific Conceptual Framework

### Framework Structure and Training Progression

The framework organizes VR-based physical AI training into 5 linked elements, summarized in Table 1. Figure 1 summarizes the proposed architecture and its feedback loops. In this framework, progressive fidelity refers to the structured increase of environmental and behavioral complexity across training tiers; staged autonomy refers to calibrating the robot’s permitted actions to clinical risk; and governed transfer refers to treating simulation-to-real progression as a gated clinical-governance decision rather than a purely technical step. The 5 elements proceed sequentially but include feedback loops: nurse-in-the-loop corrections at each fidelity stage feedback to refine the task library and role-boundary map, while digital twin synchronization provides continuous environmental grounding across all training stages. The governed transfer element enforces stage-gate progression, ensuring that advancement from simulation to physical deployment is conditioned on explicit nursing-relevant validation criteria rather than technical metrics alone.

**Table 1.** The 5 linked elements of the nurse-in-the-loop, site-specific framework, with the purpose and core mechanism of each.

Element	Purpose	Core mechanism and output
Nursing task library and role-boundary map	Define which nursing tasks are appropriate for bounded robotic support and at what level of autonomy	Decomposes nursing into task families by safety stakes, paired with a 4-zone boundary map: independent execution, nurse-confirmed execution, gated expansion, and permanently prohibited action
Nurse-in-the-loop VR^a^ training	Capture expert nursing judgment as the primary training signal rather than treating nurses as end users	Inside VR, nurses demonstrate tasks, annotate context, correct trajectories, label unsafe actions with explanations, and set escalation thresholds, yielding multimodal input richer than conventional labeled datasets
Progressive simulation fidelity	Build robot competence incrementally and avoid premature exposure to clinical complexity	Structured low-fidelity, midfidelity, and high-fidelity tiers, with promotion to a higher tier contingent on demonstrated performance at the current tier
Site-specific digital twin synchronization	Adapt generic robot capabilities to the physical and operational realities of one clinical unit	A continuously synchronized operational model constrained to variables that materially affect task performance: room geometry, equipment, circulation pathways, staffing density, and workflow conventions
Governed simulation-to-real transfer and evaluation	Condition physical deployment on nursing-relevant validation rather than on technical metrics alone	Staged validation gates, each with explicit sign-off and mandatory regression on degradation, evaluated against workload relief, nurse trust, and care quality

a VR: virtual reality.

**Figure 1.**
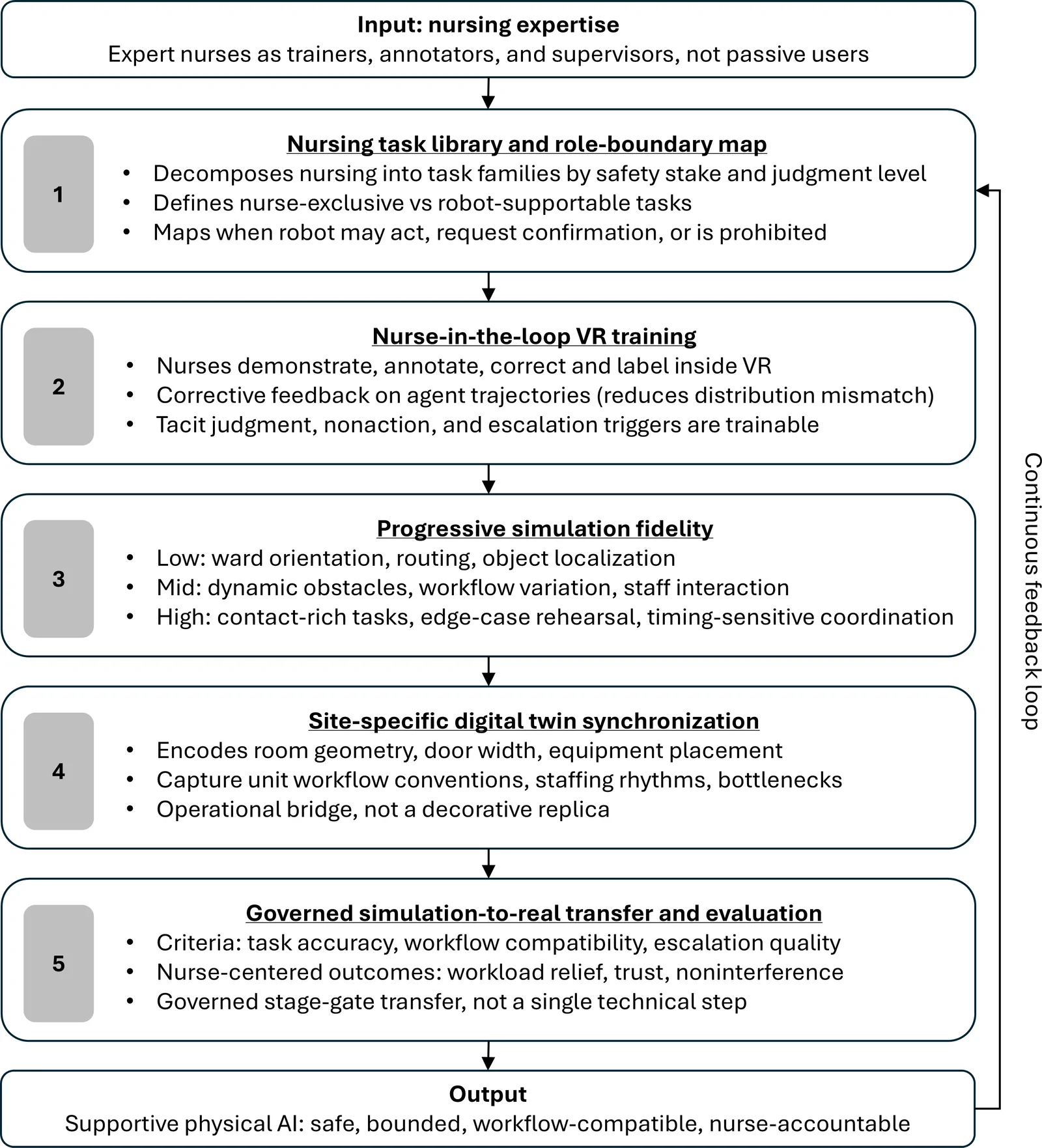
Nurse-in-the-loop, site-specific conceptual framework for virtual reality (VR)–based physical AI training in nursing.

### Nursing Task Library and Role-Boundary Map

Rather than treating “nursing” as a single training domain, the framework decomposes nursing into task families with different safety stakes, data needs, and permissible autonomy levels. This decomposition is essential because the spectrum of nursing work ranges from logistical tasks with minimal patient contact (eg, supply transport between a storage room and a nurses’ station) to high-acuity interventions requiring moment-to-moment clinical judgment (eg, responding to hemodynamic instability). Treating these as a single training domain would either constrain the robot to trivial functions or expose patients to premature autonomy in high-stakes situations. Near-term supportable tasks include supply transport, room-to-room delivery, retrieval of noncontrolled materials, and bounded monitoring-support workflows such as periodic vital sign equipment checks. The framework’s clinical scope is delimited accordingly: it is confined to supportive tasks—noncontact logistical and environmental tasks in its initial scope, with possible extension to direct physical assistance such as patient repositioning support only under the staged validation gates—and it permanently excludes surgical or otherwise invasive intervention, medication administration, and independent clinical judgment, including autonomous decisions based on the interpretation of physiological signals, all of which remain with the nurse and clinical team. The task library is paired with a role-boundary map that defines 4 zones of permissible action: independent execution (low-risk, logistical tasks with no patient contact), nurse-confirmed execution (tasks requiring verification before action, such as delivering items to a specific patient room), gated expansion (tasks involving direct physical assistance, such as patient repositioning support, which fall outside the autonomy permitted at the framework’s initial stage and may be introduced only progressively under the staged validation gates described in layer 4), and permanently prohibited action (medication administration and independent clinical decision-making, which remain reserved to the nurse at all stages). This graduated boundary structure ensures that autonomy is not a binary on or off switch but a spectrum aligned with clinical risk, in which direct-contact assistance is neither categorically excluded nor permitted at the outset but is gated behind staged, nurse-governed validation.

### Nurse-in-the-Loop VR Training

Nurses are expert contributors to policy formation, not passive end users. This distinction is not merely rhetorical; it determines the entire data collection and learning paradigm. In VR, nurses serve multiple roles simultaneously: they demonstrate tasks by performing them in the simulated environment; annotate contextual factors that affect task execution (eg, “this corridor is typically congested at shift change”); correct robot trajectories when the agent’s behavior deviates from acceptable practice, label actions as unsafe or inappropriate with explanations; and define the escalation thresholds that determine when the robot should pause and request human guidance. This multimodal expert input is substantially richer than the labeled datasets typically used in supervised robot learning, because it captures not only what to do but also why, when, and under what conditions actions are appropriate or inappropriate. The approach is consistent with interactive imitation learning, which demonstrates that corrective feedback on agent trajectories in the agent’s own state distribution reduces compounding errors more effectively than offline demonstration alone [8]. It also aligns with human-in-the-loop optimization research showing that iterative human participation in the learning loop produces applied robotic systems that better match real-world task requirements [9].

### Progressive Simulation Fidelity

Fidelity is matched to learning objectives rather than maximized indiscriminately. A common assumption in simulation-based training is that higher fidelity is always better; however, this is not supported by the evidence. Excessively complex environments can impede early-stage learning by introducing irrelevant variation before foundational competencies are established. This staged approach draws on the scaffolding principle in learning theory, in which task complexity is incrementally increased as competence develops [10]. Applied here, the robot’s training environment progresses through structured tiers of environmental and behavioral complexity, with promotion to a higher-fidelity tier contingent on demonstrated performance at the current tier. This prevents the robot from encountering clinical complexity it is not yet equipped to handle. Meta-analyses confirm that structured VR environments improve knowledge acquisition, skill development, and problem-solving in nursing education [11-14], and the same principle applies to robot training: structured progression through complexity produces more robust and generalizable policies than exposure to full complexity from the outset. The 3 fidelity tiers are defined as follows: low fidelity: ward orientation, routing, object localization, and collision avoidance; mid fidelity: dynamic obstacles, workflow variations, staff interactions, and monitoring support; high fidelity: contact-rich assistance, edge-case rehearsal, and timing-sensitive coordination.

### Site-Specific Digital Twin Synchronization

In this framework, the digital twin is defined as a continuously synchronized operational model of a specific nursing unit, constrained to the variables that materially affect robotic task performance, rather than a comprehensive, photorealistic, or static replica. It is an operational bridge, not a decorative replica. The distinction matters because health care digital twin implementations vary enormously in scope and ambition, from comprehensive hospital-wide models to narrowly scoped process simulations. A recent National Academies report identifies foundational research gaps in digital twin validation, interoperability, and domain-specific adaptation, cautioning that the term is frequently applied to systems that lack the bidirectional synchronization and predictive capability that define a true twin [15]. Health care–specific reviews note similar definitional variability and largely partial implementations [16-18]. The pragmatic approach adopted here constrains the twin to the minimum set of variables that materially affect robotic task performance in a specific nursing unit: room geometry and doorway clearances, equipment locations and dimensions, circulation pathways and bottlenecks, staffing density patterns, and unit-specific workflow conventions such as supply routes and shift-change traffic patterns. This constraint is deliberate. An overly ambitious twin risks becoming an engineering project in itself, diverting resources from the core research questions. A pragmatically scoped twin, by contrast, serves its intended function—bridging the gap between a generic simulation and the particular physical and operational realities of the deployment site—without requiring comprehensive hospital informatization as a prerequisite.

The level of fidelity a twin requires is determined by what the task depends on. Supportive tasks, such as navigation, transport, retrieval, and environmental monitoring, depend on spatial geometry, circulation patterns, equipment placement, and workflow timing, all of which the pragmatic twin represents; they do not depend on patient-specific anatomy or tissue behavior. Surgical tasks, by contrast, depend on patient-specific anatomical structure, tissue deformation, and submillimeter spatial accuracy, which is why surgical applications require high-fidelity, patient-specific twins. The pragmatic twin is therefore not an oversimplified version of a surgical twin but a different object matched to a different task class: for supportive nursing tasks it is sufficient, whereas for surgery it would be inadequate. Framing twin fidelity as task-determined, rather than as a single scale of ambition, clarifies why the scoping adopted here is appropriate rather than simplistic.

### Governed Simulation-to-Real Transfer and Evaluation

Transfer criteria extend beyond task success to include workflow compatibility, interruption recovery, escalation quality, and noninterference with bedside care. In conventional robotics, simulation-to-real transfer is treated primarily as a technical challenge: closing the gap between simulated and physical dynamics through domain randomization [19], dynamics randomization [20], and system identification. These techniques remain essential and are incorporated into the framework’s technical architecture (Multimedia Appendix 1). However, in a nursing context, the transfer gap is not only physical but also social, organizational, and ethical. A robot that navigates a physical ward successfully may still fail if it disrupts nurse workflow, creates alarm fatigue through excessive escalation, or undermines patient trust through inappropriate proximity or timing. Therefore, simulation-to-real transfer is treated here as a governance event rather than a final technical step. Transfer proceeds through staged validation gates, each requiring explicit sign-off before progression. This stage-gate structure ensures that technical performance is necessary but not sufficient for clinical deployment; nursing-relevant outcomes, including measurable workload relief, nurse trust as assessed by validated human-robot interaction scales, and care quality indicators, are the ultimate evaluation standard.

## Conceptual Technical Architecture

### Four-Layer Technical Architecture

The framework is grounded in a 4-layer technical architecture (Figure 2), with illustrative implementation options. The conceptual reference architecture, including evaluation logic and architectural requirements for each layer, is provided in Multimedia Appendix 1. Throughout this section, the contribution lies in the layered functional structure of each layer rather than in any specific product or method; all named technologies are current illustrative instantiations that satisfy a layer’s functional requirements and may be substituted by implementing teams as the tooling landscape evolves.

**Figure 2.**
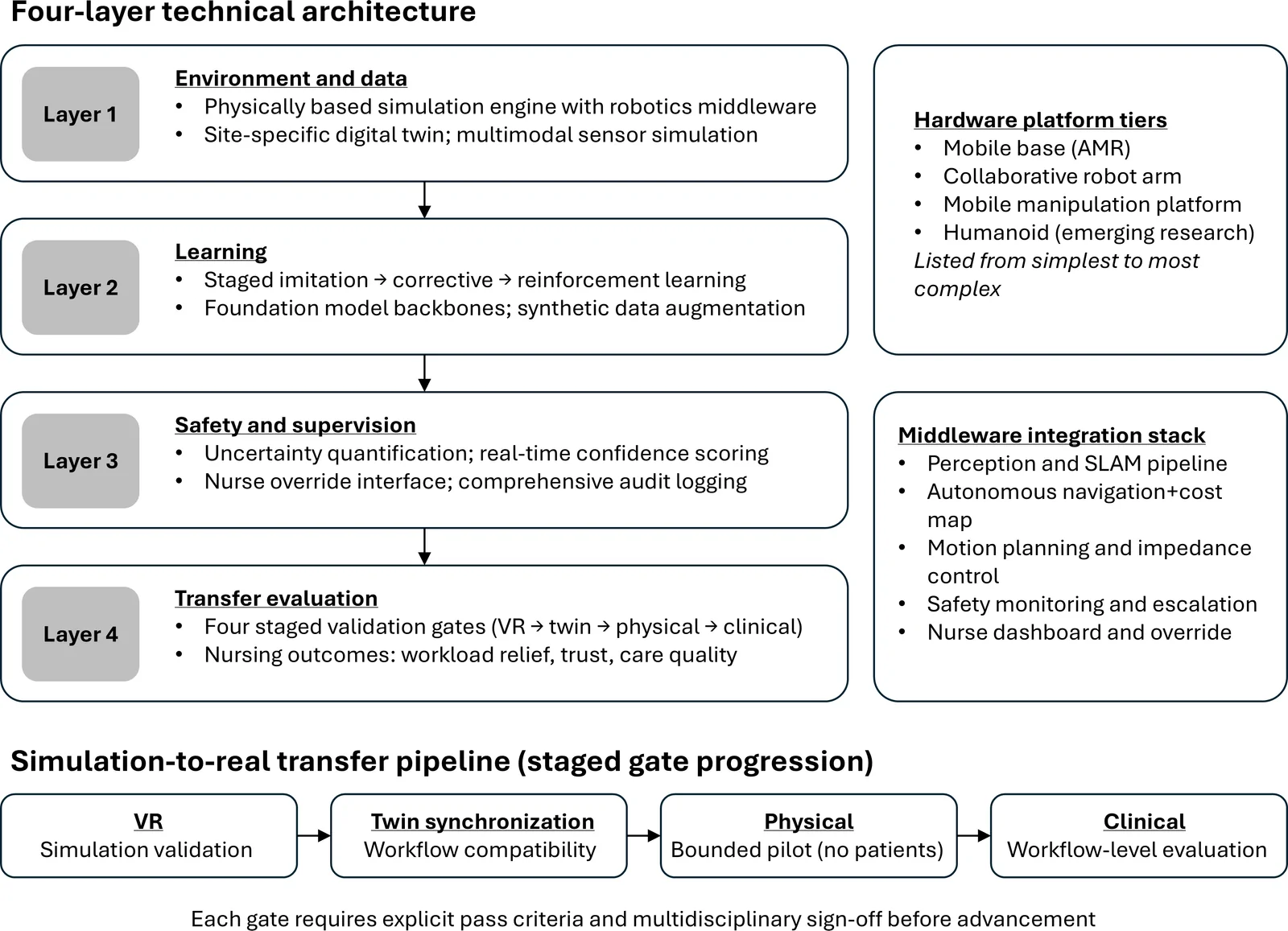
Four-layer technical architecture and simulation-to-real transfer pipeline for virtual reality (VR)–based physical AI training in nursing. AMR: autonomous mobile robot; SLAM: simultaneous localization and mapping.

### Layer 1: Environment and Data

The VR environment requires graphics processing unit–accelerated, physically based simulation with direct interoperability with robotics middleware, specifically Robot Operating System 2 (ROS 2); platforms such as Isaac Sim or Unity with a ROS 2 bridge are illustrative options that meet this requirement. For example, Isaac Sim provides native integration with a physics engine supporting both rigid and deformable body dynamics—useful for simulating clinical equipment interactions—together with built-in synthetic data generation. These are cited as representative current tools rather than as required choices; the functional requirement, not the specific platform, is what the framework specifies. Site-specific digital twins are constructed via light detection and ranging scanning or photogrammetry of target clinical units, with the resulting 3D environments synchronized with operational workflow data drawn from structured observation and, where available, real-time locating system feeds. Multimodal sensor channels are simulated within the environment to match the sensor configuration of the target physical robot platform. For the noncontact logistical and environmental tasks that constitute the framework’s initial scope, the active sensing channels are red, green, blue, and depth cameras, light detection and ranging, and inertial measurement units. Force and haptic channels are additionally represented in simulation to preserve fidelity with contact-capable platforms, including collaborative-arm and emerging humanoid configurations, which increasingly incorporate fingertip tactile and joint-level force-torque sensing as standard equipment. Because simulation-to-real transfer requires the simulated sensor suite to mirror the deployed platform, these contact channels are not engaged as policy inputs for noncontact tasks; they become active only if and when the framework is extended to contact-rich assistance under the staged validation gates (layer 4). Expert demonstrations and corrective feedback are captured as structured task-state logs, including joint positions, end-effector poses, gaze direction, verbal annotations, and contextual state labels.

### Layer 2: Learning

The learning layer operates in 3 sequential phases, each defined by its functional role rather than by a specific algorithm. First, foundation policy formation (eg, behavioral cloning from expert nurse demonstrations) provides a policy that captures the basic structure of each task. Second, interactive correction, using methods such as dataset aggregation (DAgger) or intervention-weighted regression, refines this policy by incorporating nurse feedback on the robot’s own attempted trajectories, weighting corrections by intervention timing and confidence to address the distribution mismatch problem inherent in offline imitation learning [21]. Third, policy optimization (eg, reinforcement-learning fine-tuning with algorithms such as proximal policy optimization or soft actor-critic under nursing-specific reward shaping) optimizes the policy for task completion, safety margins, workflow timing, escalation quality, and trajectory smoothness. Pretrained robot foundation-model backbones, such as OpenVLA [22] or Octo [23], may be leveraged to reduce data requirements and improve generalization across task families. The named methods are representative instances of each functional role and may be substituted as the field advances. Synthetic data augmentation using domain randomization and world foundation models expands coverage of rare but safety-critical clinical edge cases, such as unexpected patient falls, equipment malfunctions, or crowded corridor scenarios, without requiring real-world exposure to these events. Importantly, reinforcement-learning fine-tuning is not proposed as a means of solving contact-rich reality-gap problems in simulation; rather, it is applied incrementally only after lower-risk policies have passed the staged validation gates and only within the constraints of supervised real-world evaluation.

### Layer 3: Safety and Supervision

The safety layer crosscuts all other layers and operates at multiple timescales. At the real-time level, uncertainty quantification via Monte Carlo dropout or conformal prediction generates continuous confidence scores for the robot’s actions and perceptions. Hard stop rules trigger immediately when force thresholds are exceeded, when confidence drops below task-specific thresholds (τ), or when the robot detects an unrecognized situation outside its training distribution. A nurse override API, implemented as a ROS 2 action server, allows real-time human intervention with a target response latency of less than 500 milliseconds. At the session level, all decisions, confidence scores, escalation events, and nurse override instances are logged in a structured format for post hoc audit. These logs serve dual purposes: they provide the audit trail necessary for institutional safety review, and they generate the training signal for policy refinement in subsequent learning iterations. The safety layer embodies the principle that the robot should fail conservatively: when uncertain, it should stop and escalate rather than proceed with a potentially harmful action.

### Layer 4: Transfer Evaluation

Transfer proceeds through 4 stage gates, each with explicit pass or fail criteria and required sign-off before advancement. Gate 1 validates VR against task accuracy, collision rate, and escalation quality benchmarks. Gate 2 validates in the digital-twin–synchronized environment for workflow compatibility, route efficiency, and timing alignment with shift-level task patterns. Gate 3 involves bounded physical pilot deployment in a controlled clinical area without patients, evaluating physical task performance, nurse override latency, and usability. Gate 4 extends to workflow-level evaluation in a real clinical unit under supervision, integrating nursing-relevant outcomes, including measurable workload relief (NASA–Task Load Index [NASA-TLX]), nurse trust (validated human-robot interaction trust scales), and care quality indicators. No gate may be bypassed, and regression to an earlier gate is required if performance degrades at any stage. A formal multidisciplinary review, including nursing leadership, biomedical engineering, and safety officers, is required before advancing from Gate 3 to Gate 4. Within each gate, advancement is conditioned on predefined quantitative criteria of 2 kinds: absolute safety constraints that must not be violated, such as avoidance of collisions and unsafe proximity, and relative performance criteria benchmarked against acceptable task execution, such as task-completion accuracy and escalation appropriateness. Because the appropriate numeric thresholds depend on the task family, clinical unit, and risk profile, they are established and calibrated empirically during validation rather than fixed as universal constants at the conceptual stage; Multimedia Appendix 1 specifies the gate environments, evaluation-criteria categories, and nursing-relevant indicators against which these thresholds are set.

The conceptual reference architecture, including illustrative platform comparisons, sensor requirements, middleware integration logic, and a staged validation structure, is provided in Multimedia Appendix 1.

## Ethical, Professional, and Practice Implications

Governance should be built into the training pipeline from the outset, not retrofitted after technical development is complete. This means nurses should define the task library, escalation logic, and performance criteria, rather than only provide end-stage user feedback on systems designed without their input. The ethical stakes are significant: a robot operating in a clinical environment has the potential to affect patient safety, nurse workload, and the therapeutic relationship between nurse and patient. The framework addresses these stakes through several mechanisms. First, it preserves nurse authority over all ethically consequential decisions by design; the role-boundary map explicitly prohibits robotic action in domains requiring clinical judgment. Second, robotic support is bounded to clearly delegable task families where the consequences of error are manageable and reversible. Third, VR carries a governance function alongside its training function: it provides a predeployment space where action boundaries, error modes, and workflow consequences can be examined without risk to patients [5,6,24,25]. Fourth, the staged transfer gates embed ethical review into the development pipeline, ensuring that the decision to deploy a robot in a clinical unit is made by a multidisciplinary team, rather than by the technical development team alone. These design choices reflect a broader commitment to the principle that AI in nursing should augment professional practice rather than displace professional judgment [5,6].

Beyond these design commitments, 3 areas require explicit treatment because they condition whether such a system can be responsibly developed and deployed. The first concerns regulatory classification and legal responsibility. A learned policy that supports nursing tasks may, depending on its function and claims, fall within medical-device-software regulation, such as the US Food and Drug Administration framework for Software as a Medical Device [26,27], and, in other jurisdictions, within risk-based regimes such as the European Union Artificial Intelligence Act [28], both of which impose validation, documentation, and postdeployment monitoring obligations that a nursing robotics program must anticipate from the outset. Closely related is the allocation of liability when an autonomous system contributes to an adverse event: responsibility may be distributed among the nurse who supervises the system, the institution that deploys it, and the developer or manufacturer that builds it, and this distribution is not yet settled in law or policy. The framework does not resolve this question, but its retained human accountability, explicit role boundaries, and multidisciplinary sign-off at each transfer gate are designed to keep responsibility traceable rather than diffuse, providing an auditable record of who authorized what at each stage. The second and third areas concern data governance and patient consent. Nurse demonstrations, corrective feedback, and deployment logs generate behavioral and environmental data, and in a clinical setting this data may incidentally capture patient-related information; such data must be handled under institutional governance consistent with health-privacy law, including, in the United States, HIPAA-consistent deidentification and the exclusion of patient-identifiable information from training corpora. A distinct and less frequently acknowledged question is the ownership of and consent for the nurses’ tacit expertise that the framework deliberately elicits and encodes, because the nurse-in-the-loop paradigm treats expert judgment as a primary training signal, the professionals whose knowledge is captured have a legitimate interest in how that knowledge is used, attributed, and governed. Finally, patients have an interest in robotic support operating in their care environment even when tasks are noncontact; institution-level notice-and-consent processes should make patients aware of robotic support and provide a means to decline it, preserving patient autonomy as a first-class consideration rather than an afterthought. Treating these regulatory, data-governance, and consent questions as design requirements, rather than as compliance steps addressed after development, is consistent with the framework’s commitment to building governance into the pipeline from the outset.

## Discussion

This paper repositions VR from a visualization tool to a translational training infrastructure connecting nursing expertise with robotic execution. The claim is not that nursing requires an entirely separate technological universe from big-tech physical AI; rather, nursing needs an additional nurse-centered orchestration layer that specifies workflow fit, role clarity, safety governance, and local adaptation as nonnegotiable adoption criteria. This complements the call by Shaw and Chen [3] for nursing-specific engagement with physical AI by providing a concrete architectural pathway from concept to implementation.

The 4-layer technical architecture grounds the framework in concrete implementation considerations. By specifying graphics processing unit–accelerated simulation, robotics middleware integration, staged imitation-to-reinforcement learning, foundation model backbones, uncertainty quantification, and staged transfer gates, the framework provides an implementation-aware roadmap that connects conceptual nursing robotics discourse with concrete implementation pathways. Each architectural layer addresses a specific translational challenge: layer 1 bridges the gap between clinical environments and simulation; layer 2 structures the learning process around nurse expertise rather than autonomous exploration; layer 3 embeds safety governance into real-time operation; and layer 4 enforces validation against nursing-relevant outcomes before any clinical contact occurs. This layered structure is intended to make the framework’s plausibility conceptually arguable and incrementally verifiable through independently testable stages, rather than being assumed.

The strategic contribution is clarity of scope and sequence. Rather than proposing a generalized nursing humanoid—an aspiration that outpaces both current technology and clinical readiness—the framework defines a tractable developmental pathway: build a prototype VR environment for bounded supportive nursing tasks, test nurse-in-the-loop data collection and correction workflows, synchronize with one real clinical unit, and evaluate transfer against nursing-relevant outcomes. This incremental approach is more clinically defensible because it allows each stage to be independently validated and because it confines early-stage risk to simulation rather than exposing patients or staff to insufficiently tested systems. It also aligns with how nursing practice itself manages complexity: through structured protocols, graduated responsibility, and continuous reassessment.

Several features distinguish this framework from existing approaches to health care robotics. Most current health care robots are developed outside nursing and offered to nurses as finished products; this framework places nurses at the center of the development process from task selection through policy training to transfer evaluation. Most simulation-to-real pipelines in robotics treat transfer as a technical challenge to be solved through domain randomization; this framework treats transfer as a governance event requiring nursing-relevant validation. Further, most health care digital twin implementations aspire to comprehensive hospital models; this framework adopts a pragmatically scoped twin constrained to variables that materially affect robotic task performance. These distinctions are not arbitrary; they follow from the recognition that nursing’s requirements are not merely a subset of general robotics requirements but include qualitatively different demands around workflow integration, role clarity, and preserved professional authority.

This contrast is clearest when supportive autonomy is set against surgical autonomy. Surgical autonomy centers on contact-rich, submillimeter, and often irreversible manipulation of patient anatomy, which requires patient-specific high-fidelity modeling and fine force-level control. Supportive autonomy, as framed here, centers instead on logistical and environmental tasks whose errors are comparatively bounded and recoverable; where direct physical assistance is eventually introduced, it enters only under staged, nurse-governed validation gates rather than as the operating premise. This difference in kind is precisely why the framework’s architectural choices fit supportive autonomy and would be insufficient for surgery: the pragmatically scoped digital twin, the workflow-centered and role-centered validation, and the gated transfer logic are calibrated to bounded support rather than to anatomical precision. What carries across domains is not the technical stack but the governance: expert-in-the-loop policy formation, staged validation, and retained human accountability are not specific to nursing and could inform surgical contexts, even though the fidelity and precision they must achieve differ fundamentally.

The framework also raises questions that warrant further investigation. How many expert nurse demonstrations are sufficient to train a robust foundation policy for a given task family? What is the minimum viable scope of a digital twin that preserves transfer validity? How should escalation thresholds be calibrated to balance safety against workflow disruption from excessive escalation? And how do nurses’ trust and acceptance of robotic support evolve as they participate in the training process vs receiving a pretrained system? These are empirical questions that the framework is designed to enable rather than preemptively answer.

## Limitations

Five limitations warrant explicit acknowledgment. First, this paper is conceptual and does not empirically test the framework; the proposed architecture is a structured hypothesis for future empirical validation. Second, the simulation-to-real gap remains incompletely solved. Sensor noise and clinical unpredictability will create transfer challenges that simulation cannot fully anticipate, and physical contact dynamics in particular—deformable-tissue interaction and subtle force modulation—fall outside the framework’s initial noncontact scope and would require dedicated modeling and validation before any contact task could be approached. Accordingly, the transition from behavioral cloning to reinforcement learning is positioned not as a simulation step but as fine-tuning in the validated real environment: behavioral cloning establishes a safe foundation policy from expert demonstrations, while reinforcement-learning refinement is applied incrementally under the staged transfer gates rather than to anticipate contact dynamics in simulation. Multimedia Appendix 1 characterizes the principal dimensions of this transfer gap. Third, the digital twin literature remains technically immature in health care; implementers must calibrate expectations and adopt the pragmatic constraint recommended here. Fourth, hardware platform selection (autonomous mobile robot, cobot arm, and humanoid) will substantially shape which components are feasible and how the twin must be constructed. Fifth, although the framework places nurses at the center of robotic policy formation as a matter of design, this paper was itself developed through literature synthesis rather than through direct co-design with nurses, patients, or administrators. These are distinct levels of nurse centrality: positioning nurses as the authority who defines robotic scope is a property the framework specifies, whereas codeveloping that specification with practicing nurses is an empirical step the framework explicitly requires rather than one this conceptual paper performs. Accordingly, participatory co-design is not omitted but deferred as a core element of the next stage: future empirical work should begin with a participatory design phase in which nurses from the target clinical unit codevelop the task library, role-boundary map, and escalation logic before any VR environment is constructed. This sequencing—defining what nurses should govern at the conceptual stage and operationalizing that governance through participation next—resolves the apparent tension that the absence of co-design at this stage might otherwise suggest.

Future research should pursue several priorities. First, prototype VR environments should be constructed for specific bounded nursing task families, beginning with logistical tasks (supply transport and equipment retrieval) before advancing to more complex support tasks involving dynamic human interaction. Second, digital twin construction feasibility should be examined in real clinical units, including assessment of the time, cost, and institutional cooperation required to achieve adequate twin fidelity. Third, empirical studies should evaluate technical performance alongside nurse trust, workflow compatibility, and escalation quality, using mixed methods designs that combine quantitative performance metrics with qualitative analysis of nurses’ experiences. Fourth, longitudinal studies should track how nurse trust and acceptance evolve over repeated interactions with the training and deployment pipeline. Finally, cross-site generalization should be investigated: how much of the training pipeline transfers when the framework is applied to a different clinical unit with different geometry, workflow patterns, and nursing culture?

## Conclusions

Physical AI in nursing should be framed as supportive robotics that reduce repetitive and physically burdensome work while preserving nurses’ authority over judgment, prioritization, and relational care. This framing is not a concession to technological limitations; it is a principled design commitment grounded in what nursing is and what robots can meaningfully contribute to nursing practice in the near to medium term.

The nurse-in-the-loop, site-specific VR framework proposed in this paper addresses the current gap between conceptual promise and clinical feasibility through several integrated mechanisms. The task library and role-boundary map ensure that the robotic scope is defined by clinical judgment rather than technical capability. Nurse-in-the-loop VR training ensures that the robot’s learned policies reflect expert nursing knowledge, contextual awareness, and safety priorities. Progressive simulation fidelity structures the learning process to build competence incrementally, avoiding premature exposure to clinical complexity. Site-specific digital twin synchronization bridges the gap between generic simulation and the particular physical and organizational realities of the deployment environment. Governed simulation-to-real transfer ensures that advancement from simulation to physical deployment is conditioned on explicit nursing-relevant validation criteria, with human accountability retained at every stage.

Taken together, these elements constitute a translational infrastructure rather than a single technological intervention. VR is not merely a visual training aid in this framework; it is the environment where nursing expertise is elicited, structured, annotated, stress-tested, and safely transferred to physical systems. The digital twin is not a visualization tool; it is the mechanism through which generic robot capabilities are adapted to local clinical realities. Further, the nurse is not an end user to be consulted after development; the nurse is the domain expert whose knowledge, judgment, and workflow requirements drive the entire pipeline.

This framework contributes to nursing informatics by proposing a concrete mechanism through which nursing knowledge can be formally structured and transmitted to physical AI systems, extending the discipline’s engagement with AI beyond decision support and documentation into the domain of embodied, physically acting systems. It contributes to robotics by articulating a domain-specific orchestration layer that connects general-purpose physical AI capabilities with the particular requirements of a complex, safety-critical, and human-centered work environment. It also contributes to patient safety by ensuring that the path from simulation to clinical deployment is governed by explicit criteria, staged validation, and retained human authority.

The path forward requires sustained collaboration between nursing scientists, robotics engineers, clinical administrators, and the nurses who ultimately work alongside these systems. The framework proposed here is intended as a structured starting point for that collaboration—not a finished system, but a principled architecture within which empirical questions can be posed, tested, and iteratively refined.

## Supplementary material

10.2196/96848 Multimedia Appendix 1 Technical reference architecture for the proposed framework, including virtual reality environment construction, digital twin synchronization, the learning pipeline, staged simulation-to-real validation, and hardware and robotics-middleware integration.

## Abbreviations

NASA-TLX: NASA–Task Load Index
ROS 2: Robot Operating System 2
VR: virtual reality
