# Supplementary material for: Virtual Reality–Enabled Physical AI Training for Supportive Nursing Robotics: Nurse-in-the-Loop, Site-Specific Conceptual Framework
Source: JMIR Nurs. 2026 Oct 5;9:e96848. doi: 10.2196/96848 (PMC13637984; doi:10.2196/96848)
Supplement: Supplementary file 1 [file nursing-v9-e96848-s001.docx]

**Multimedia Appendix 1**

**Technical Implementation Architecture for VR-Based Physical AI Training in Nursing**

# Overview

This appendix provides the conceptual technical architecture, illustrative tool categories, and evaluation logic underpinning the conceptual framework presented in the main manuscript. It is structured around five sections corresponding to the five key technical domains: (1) VR environment construction, (2) digital twin implementation and synchronization, (3) AI/ML learning pipeline, (4) simulation-to-real transfer methodology, and (5) physical AI hardware options and ROS 2 integration. Each section describes the architectural requirements and illustrative implementation options relevant to a nursing clinical context. Specific tool selections and optimized configurations will vary by research context and are left to implementing teams.

This appendix is designed to support assessment of the framework's conceptual plausibility by peer reviewers and to provide a conceptual reference architecture for research teams planning prototype development.

**Figure A1.** Four-layer technical architecture and simulation-to-real transfer pipeline.

# Section 1: VR Environment Construction

## 1.1 Engine Selection

Two primary platforms are suitable for this application, each with distinct trade-offs for nursing robotics research:

| Criterion | GPU-Accelerated Physics Engine | Game Engine with Robotics Bridge |
| --- | --- | --- |
| Physics engine | GPU-accelerated rigid and deformable body simulation | General-purpose physics — less suited for robot dynamics |
| ROS 2 integration | Native robotics middleware bridge | Plugin-based connector (additional setup) |
| Synthetic data | Built-in synthetic data generation and world model integration | Perception-based data generation (less mature) |
| Haptic support | Via haptic SDK integration | VR platform haptic API |
| Learning frameworks | Native RL and IL framework support | Manual wrapper required for learning frameworks |
| License & cost | Free for research use | Free for educational/personal use |
| Best for | Full robotics research pipeline | Rapid environment prototyping |

A GPU-accelerated physics engine with native robotics middleware interoperability is recommended as the primary simulation platform. Game engines with robotics bridge plugins remain viable for early-stage clinical environment prototyping where faster iteration speed is prioritized over physics fidelity. The choice of engine should be guided by the team’s existing infrastructure and the fidelity requirements of the target task families.

## 1.2 Clinical Environment Reconstruction

The VR clinical environment must faithfully represent the target nursing unit. Recommended reconstruction workflow:

- 3D scanning: high-precision LiDAR scanning (sub-centimeter accuracy) for ward geometry capture
- Point cloud processing: open-source point cloud tools for mesh optimization, exported in a format compatible with the chosen simulation engine
- Equipment modeling: photogrammetry-based reconstruction of clinical equipment (IV poles, vital monitors, bed rails, medication carts)
- Dynamic elements: patient mannequin physics (articulated rigid body), clinical staff avatars (motion-captured or procedurally animated)
- Environmental fidelity: ambient noise (EHR alert sounds, hallway activity), lighting variation (day/night shift), occupancy patterns

## 1.3 Nursing Scenario Library

Clinical scenarios should be stratified by fidelity tier and validated against nursing workflow documentation. Consistent with the main manuscript, the lower tiers correspond to the framework's initial non-contact scope, whereas higher-fidelity scenarios involving direct physical assistance—such as patient repositioning—correspond to the gated-expansion zone and are introduced only under staged, nurse-governed validation; medication-related items denote delivery and transport support only, as medication administration is permanently excluded from autonomous action. The tiers are organized as follows:

| Fidelity Tier | Representative Scenarios | Key Technical Requirements |
| --- | --- | --- |
| Low | Ward navigation, supply retrieval, equipment transport | Spatial mapping, obstacle avoidance, path planning |
| Mid | Vital sign monitoring support, meal delivery, linen management | Human detection, timing logic, interruption handling |
| High | Patient repositioning assist, dressing cart preparation, medication delivery support | Force control, fine manipulation, safety-critical escalation |
| Edge Case | Fall risk detection, rapid deterioration alert, code cart retrieval | Rare event synthesis, uncertainty quantification, immediate escalation |

# Section 2: Digital Twin Implementation and Synchronization

## 2.1 Digital Twin Scope Definition

Consistent with the main manuscript's pragmatic definition, the digital twin for this framework is not a comprehensive hospital-wide model. It is scoped to variables that materially affect robotic task performance in a specific nursing unit:

- Spatial layer: room geometry, doorway clearances (per applicable accessibility standards), corridor widths, turning radii for robot platform
- Equipment layer: locations and dimensions of beds, IV poles, medication carts, vital sign monitors, call systems, hand hygiene stations
- Workflow layer: common task sequences per shift, peak traffic periods (shift change, meal times), standard supply routes
- Personnel layer: average staffing density per zone, typical nurse station occupancy, communication patterns
- Dynamic layer: real-time occupancy (optional for advanced implementations via IoT sensors)

## 2.2 Data Collection Protocol

A structured data collection protocol minimizes disruption to clinical operations while achieving sufficient twin fidelity:

| Data Type | Collection Method | Duration | Output Format |
| --- | --- | --- | --- |
| Spatial geometry | LiDAR scanning | Off-hours session | Point cloud → 3D mesh |
| Equipment inventory | Photogrammetry + inventory logging | Single session | 3D assets + spatial database |
| Workflow patterns | Structured observation (multiple shifts) | Multiple shift observations | Structured task sequence logs |
| Traffic patterns | De-identified location tracking data | Retrospective window | Heatmap + time-series data |
| IoT sensor data | Environmental sensors (optional) | Continuous (IRB-approved) | Streaming time-series database |

All data collection requires IRB approval. Patient-identifiable information must be excluded; de-identified operational data is sufficient for the spatial and workflow twin components.

## 2.3 Synchronization Architecture

The digital twin maintains synchronization with the physical ward through a three-tier update cycle:

- Real-time layer: robot position, sensor readings, active task state — via robotics middleware publish-subscribe
- Session layer (per shift): equipment positions, staffing configuration, active patient count — via API integration with clinical information systems
- Structural layer (monthly): geometry updates, equipment procurement changes — via re-scan triggered by facilities management

Recommended middleware approach: a current LTS release of a robotics middleware framework with IoT edge device support and a bridge to the simulation engine’s scene synchronization layer.

## 2.4 Twin Fidelity Validation

Before using the twin for robot training, geometric and operational fidelity should be validated:

- Geometric accuracy: compare scanner-derived mesh against manual measurements at distributed reference points; target sub-decimeter RMSE
- Navigation equivalence: run identical path planning algorithm in twin and physical ward; compare route lengths and obstacle encounter rates
- Workflow concordance: simulate representative task sequences derived from observation data; compare timing distributions against observed data using appropriate statistical tests for distributional equivalence

# Section 3: AI/ML Learning Pipeline

## 3.1 Pipeline Architecture Overview

The learning pipeline operates in three phases corresponding to the scaffolded training stages described in the main manuscript:

| Phase | Learning Method | Primary Framework | Data Source | Output |
| --- | --- | --- | --- | --- |
| Phase 1: Foundation | Behavioral cloning with online corrective feedback | Imitation learning framework | Expert nurse demonstrations | Base policy π₀ |
| Phase 2: Refinement | Intervention-weighted corrective learning | Custom simulation wrapper | Corrective nurse feedback | Refined policy π₁ |
| Phase 3: Autonomy | Policy-gradient RL fine-tuning | Staged RL fine-tuning framework | Simulated reward signals plus supervised real-world validation data under staged gates | Gate-conditioned deployment candidate policy π₂ |

Reinforcement-learning fine-tuning should be interpreted as staged, safety-constrained refinement rather than as a purely simulation-based solution to the reality gap; policies become deployment candidates only after passing the validation gates described in Section 4.3.

## 3.2 Foundation Model Backbone

Rather than training from scratch, the framework leverages pre-trained robot foundation models as policy backbones, substantially reducing data requirements and improving generalization:

| Model | Architecture | Pre-training Data | Nursing Relevance | Access |
| --- | --- | --- | --- | --- |
| Proprietary VLA model (e.g., RT-2 class) | Vision-Language-Action | Large-scale web + robot data | Language-conditioned task specification | Restricted (research only) |
| Proprietary dexterous model (e.g., π₀ class) | Flow-matching generative policy | Large-scale robot + proprietary data | Dexterous manipulation | Restricted (research agreement) |
| Open-source VLA (e.g., OpenVLA) | LLM-based action prediction | Open multi-robot dataset | Fully customizable | Open-source |
| Open-source lightweight model (e.g., Octo) | Transformer-based policy | Open multi-robot dataset | Lightweight, suitable for real-time inference | Open-source (permissive license) |

The choice of foundation model backbone should prioritize open licensing and reproducibility. At the time of writing, openly licensed models offer sufficient capability for initial prototype development, while proprietary models with restricted access may be explored as optional enhancements. The foundation model landscape is evolving rapidly; implementing teams should evaluate the current state of available models at the time of development.

## 3.3 Reward Function Design for Nursing Tasks

RL fine-tuning requires nursing-relevant reward functions that go beyond task completion metrics:

| Reward Component | Formulation | Weight | Rationale |
| --- | --- | --- | --- |
| Task completion | Binary success/failure | Highest | Primary objective |
| Safety margin | Distance-based penalty for proximity violations | High | Patient and staff safety |
| Workflow timing | Time-window-based reward | Moderate | Nursing workflow fit |
| Escalation quality | Reward for appropriate uncertainty detection and escalation; penalty for false alarms | Moderate | Appropriate autonomy boundaries |
| Smoothness | Trajectory smoothness optimization | Low | Patient comfort, professional appearance |

## 3.4 Software Stack Summary

The software environment should be built on a current Linux LTS distribution with a robotics middleware LTS release, a GPU-accelerated simulation engine, a mainstream deep learning framework with imitation learning and reinforcement learning libraries, and containerization for reproducibility. Specific version selections should be made at the time of implementation to ensure compatibility and long-term support.

# Section 4: Simulation-to-Real Transfer Methodology

## 4.1 Transfer Gap Analysis

The simulation-to-real gap in nursing robotics manifests across four dimensions, each requiring specific mitigation strategies:

| Gap Dimension | Manifestation in Nursing Context | Mitigation Strategy |
| --- | --- | --- |
| Dynamics gap | Simulated patient weight/resistance differs from real; IV pole friction varies | Systematic dynamics randomization; physical system identification |
| Visual gap | Lighting variation, glare on medical equipment surfaces, clinical attire diversity | Domain randomization across visual parameters; simulation-to-real visual adaptation techniques |
| Behavioral gap | Real nurses and patients respond differently than NPC avatars | High-quality human behavior models; confederate participants in pilot; Wizard-of-Oz testing |
| Workflow gap | Actual task sequencing deviates from scripted scenarios | Multi-scenario training; distribution broadening via synthetic workflow variation; nurse-in-the-loop correction |

## 4.2 Domain Randomization Parameters

Systematic domain randomization is applied across multiple dimensions during training to improve policy robustness at deployment. Randomization should cover physics parameters (object mass, friction, damping), sensing conditions (camera exposure, sensor noise profiles), environmental variation (lighting, object placement, floor textures), human proxy behaviors (walking speed, trajectory variance, response timing), and task parameters (object orientation, quantities, arrival timing). The specific randomization ranges should be calibrated empirically during development to balance training diversity against policy convergence.

## 4.3 Staged Transfer Validation Protocol

Transfer proceeds through four explicit validation gates before clinical deployment:

| Gate | Environment | Pass Criteria | Nursing-Specific Metrics |
| --- | --- | --- | --- |
| Gate 1: VR Validation | Primary simulation environment | Task success, collision rate, and escalation precision meet pre-defined benchmarks | Appropriate escalation in scripted uncertainty scenarios above threshold |
| Gate 2: DT-Sync Validation | Digital twin of target ward | Route efficiency within acceptable deviation of optimal; timing compatible with shift workflow windows | No interference with scripted nurse workflows across repeated simulation runs |
| Gate 3: Physical Pilot | Controlled lab or empty ward (no patients) | Physical task success above threshold; reliable safe stop on contact; nurse override within real-time latency requirements | Nurse usability rating above acceptance threshold (adapted HRI usability scale) |
| Gate 4: Clinical Evaluation | Bounded real ward deployment (IRB, supervision) | Workload reduction (validated workload assessment); no adverse safety events | Nurse trust (validated HRI trust instrument); care quality indicators (process measures) |

## 4.4 Failure Mode Analysis

Prior to each transfer gate, a structured Failure Mode and Effects Analysis (FMEA) should be conducted covering:

- Sensor failure modes: RGB-D occlusion, LiDAR saturation from reflective surfaces, IMU drift during extended operation
- Actuation failure modes: motor stall, gripper slip, wheel slip on clinical flooring (vinyl, tile transitions)
- Decision failure modes: false confidence (overcommitment), false uncertainty (excessive escalation), misidentified patient state
- Human interaction failure modes: unexpected patient movement, nurse instruction ambiguity, crowded corridor navigation failure

FMEA findings must be documented and addressed before progression to the next gate. Unmitigated high-severity failures are a gate blocker. The transition from Gate 3 (controlled physical pilot) to Gate 4 (bounded clinical deployment) is the most ethically and regulatory-sensitive step in the pipeline. This transition should be governed by a formal Go/No-Go milestone review conducted by a multidisciplinary committee including the PI, nursing unit leadership, biomedical engineering, infection control, and the institutional IRB liaison. The committee should review Gate 3 performance data, FMEA residual risk documentation, and nurse usability feedback before authorizing clinical deployment.

# Section 5: Physical AI Hardware Options and ROS 2 Integration

## 5.1 Hardware Platform Selection

Three hardware platform tiers are recommended, aligned with the project timeline and task scope described in the main manuscript:

| Platform | Representative Systems | Appropriate Task Scope | Relative Cost | Development Phase |
| --- | --- | --- | --- | --- |
| Autonomous Mobile Robot (AMR) | Research-grade mobile base with optional arm attachment (various vendors) | Supply transport, fetching, ward navigation, environmental monitoring | Low | Initial prototype |
| Collaborative Robot Arm (Cobot) | 6-7 DOF collaborative robot arm with force sensing (various vendors) | Equipment handling, dressing cart prep, medication delivery support, reach assist | Moderate | After navigation tasks validated |
| Mobile Manipulation Platform | Integrated mobile manipulation platform (various vendors) | Combined navigation + manipulation; broader nursing support scope | Moderate–High | After component tasks validated |
| Humanoid (Research) | Research humanoid platform (emerging category, various vendors) | Full nursing support scope; complex patient interaction | High | After platform maturation |

For an initial research prototype, the AMR tier with an optional collaborative arm attachment is recommended. This tier provides sufficient capability for supply transport and limited manipulation tasks while remaining within typical research budget constraints.

## 5.2 Sensor Integration Specifications

| Sensor Type | Recommended Hardware | Specification | Role in Framework |
| --- | --- | --- | --- |
| RGB-D Camera | Stereo depth camera (×2) | Suitable depth range, field of view, and frame rate for indoor clinical environments | Object detection, patient state estimation, workspace mapping |
| LiDAR | Multi-channel LiDAR | 360° horizontal coverage with sufficient range for indoor navigation | Navigation SLAM, occupancy mapping, human detection |
| IMU | High-precision IMU | Sub-degree static accuracy; high update rate | Localization, fall detection, base stability |
| Force/Torque | Wrist-mounted force/torque sensor | High-frequency sampling with sub-Newton resolution | Contact safety and manipulation feedback for the gated-expansion (contact) stage; not engaged as a policy input during the initial non-contact tasks |
| Microphone Array | Multi-microphone array | Omnidirectional beamforming capability | Nurse voice command recognition, patient distress detection |

## 5.3 ROS 2 Integration Architecture

The complete ROS 2 software architecture connecting VR training, digital twin synchronization, and physical robot deployment:

- Core middleware: robotics middleware (current LTS release) with DDS-based communication; QoS profiles tuned for real-time control requirements
- Perception stack: depth image processing pipeline feeding into a SLAM-based mapping and localization system
- Navigation stack: behavior-tree-based navigation with a custom nursing-aware costmap incorporating patient proximity zones and dynamic footprint for corridor navigation
- Manipulation stack: motion planning with hardware abstraction and Cartesian impedance control for safe patient-proximate operation
- Learning policy bridge: policy inference node subscribing to robot state and publishing velocity and joint trajectory commands
- Safety layer: confidence score monitoring with emergency stop and escalation request capabilities; watchdog timer for liveness verification
- Nurse interface: real-time override action server with a web-accessible robot status dashboard
- Data logging: comprehensive topic recording with structured export for the ML pipeline; HIPAA-compliant de-identification before storage

# Appendix Conclusion

This appendix provides a conceptual technical foundation for the nurse-in-the-loop VR framework described in the main manuscript. The architectural decisions—GPU-accelerated simulation, robotics middleware integration, staged imitation-to-reinforcement learning, foundation model backbones, domain randomization, and four-gate transfer validation—are grounded in current best practice in robotics and human-in-the-loop learning, adapted for the nursing clinical context.

The architecture is intentionally modular. Research teams may substitute individual components at each architectural layer (e.g., alternative simulation engines, different robot platforms, or other learning frameworks) without invalidating the overall framework. The four-layer structure and five-element conceptual framework remain intact across hardware and software variations.

Together, the main manuscript and this appendix provide a conceptual-to-architecture package: the paper contributes the theoretical framework, while this appendix illustrates the conceptual plausibility and implementability of the framework at the architectural level. Detailed implementation specifications, optimized configurations, and empirical validation protocols will be developed as part of future prototype research.
